# The RECONCILE study protocol: Exploiting image-based risk stratification in early prostate cancer to discriminate progressors from non-progressors (RECONCILE)

**DOI:** 10.1371/journal.pone.0295994

**Published:** 2024-10-17

**Authors:** Teresa Marsden, Gerhardt Attard, Shonit Punwani, Francesco Giganti, Alex Freeman, Aiman Haider, Anna Wingate, Norman Williams, Tom Syer, Nora Pashayan, Caroline M. Moore, Mark Emberton, Clement Orczyk

**Affiliations:** 1 UCL Division of Surgical & Interventional Science, University College London, London, United Kingdom; 2 Department of Urology, University College London Hospitals NHS Foundation Trust, London, United Kingdom; 3 Cancer Institute, University College London, London, United Kingdom; 4 Centre for Medical Imaging, University College London, London, United Kingdom; 5 Department of Radiology, University College London Hospitals NHS Foundation Trust, London, United Kingdom; 6 Department of Pathology, University College London Hospitals NHS Foundation Trust, London, United Kingdom; 7 Department of Applied Health Research, University College London, London, United Kingdom; ARNAS Civico Di Cristina Benfratelli: Azienda Ospedaliera di Rilievo Nazionale e di Alta Specializzazione Civico Di Cristina Benfratelli, ITALY

## Abstract

**Introduction:**

RECONCILE (ClinicalTrials.gov:NCT04340245) will identify molecular and radiomic markers associated with clinical progression and radiological progression events in a cohort of localised, newly diagnosed Gleason 3 + 4 tumours. Molecular markers will be correlated against standard of care MRI-targeted histology and oncological outcomes.

**Methods:**

RECONCILE is an ethics approved (20/LO/0366) single centre, prospective, longitudinal, observational cohort study of recently diagnosed (within 12 months), organ-confined Gleason 3 + 4 cancers (MCCL ≤10mm) currently under active surveillance. 60 treatment-naïve participants with a concordant MRI lesion (Likert score 4 or 5) and PSA ≤ 15 ng/ml will be recruited.

Blood, urine and targeted prostate tissue cores will be subject to next generation sequencing at baseline and one year in all participants. Semen will be collected from a specified sub-population. Baseline and interval MR images will be extracted from standard of care prostate MRI ahead of radiomic analysis. Data extracted from radiological and biological samples will be used to derive the association of molecular change and radiological progression, the primary outcome of the study.

To compensate for spatial intratumoral heterogeneity and inherent sampling bias, a molecular index will be derived for each participant using the molecular profile of tumour tissue at both baseline (MolBL) and one year (MolFU). We will extract a ΔMolBL:MolFU score for each participant. Molecular progression will be defined as a MolBL:MolFU score >95% CI of the combined ΔMolBL scores. Radiological progression is defined as a PRECISE score of 4 or 5. The study is powered to detect an association with a statistical power of 80%.

**Results:**

Recruitment began in July 2020 (n = 62). To date, 37 participants have donated tissue for analysis.

**Conclusion:**

We have designed and implemented a prospective, longitudinal study to evaluate the underlying molecular landscape of intermediate risk, MR-visible prostate tumours. Recruitment is ongoing.

## 1. Introduction

Prostate cancer is the most common cancer in men and second most common cause of male cancer-related death in the UK [1]. There is discrepancy between its high incidence and low mortality rate, and so far, we have been unable to accurately identify cancers which will benefit from early treatment, and those which can be safely surveyed. Prospective randomised controlled trials have demonstrated no impact on disease specific survival when curative intervention is delayed using active surveillance and have called into question the clinical significance of Gleason 3 + 4 disease, which forms the bulk of incidentally detected prostate cancers [2,3].

The RECONCILE study will follow a cohort of newly diagnosed multiparametric magnetic resonance imaging (MRI) visible Gleason 3 + 4 prostate tumours over a one-year period of standard of care active surveillance. The cohort will be deeply characterised using radiomic, tissue and fluidic analysis at baseline and one year. Molecular and radiomic markers associated with radiological and clinical progression events will be extracted from research samples and used to derive the association of molecular change and radiological change (in particular radiological progression), the primary outcome of the study. Markers will be correlated against standard of care MRI-targeted histology and oncological outcomes to further risk stratify this cohort of intermediate risk disease at the time of baseline diagnosis.

### 1.1 Background and rationale

Long term follow-up of localised prostate cancer cohorts suggests limited benefit of radical intervention. Twenty-nine year follow up within the SPCG-4 trial suggests a low survival benefit of an additional 2.3 years (1 in every 8.4 treated) in those randomised to radical prostatectomy vs watchful waiting [2]. The absolute benefit associated with radical prostatectomy increased by a factor of more than 2 between 10 and 23 years of follow-up for both overall, and disease-specific mortality. The time taken to observe a substantial benefit from treatment suggests the need to carefully select those who may benefit from early intervention and to consider deferring curative treatment in those with lower-risk disease. Subgroup analysis also suggested the risk of death associated with Gleason 3 + 4 disease to be similar that of low-grade Gleason 6 tumours, calling into question the benefit of early, radical intervention in this cohort [2].

The UK ProtecT trial similarly showed encouraging results for men with localised cancers wishing to pursue surveillance in the first instance [3]. The study randomised patients with prostate specific antigen (PSA) screen detected cancers to surgery, radiotherapy, or active surveillance and found no difference in survival between radical treatment and monitoring groups at 10 years. Transition to radical treatment was permitted in the monitoring arm in case of clinical and biological progression Surveillance was therefore considered a safe option for men with localised disease even before the era of MRI-led diagnosis [4].

Multiparametric MRI now forms the cornerstone of localised prostate cancer diagnosis and is recommended prior to diagnostic biopsy in several clinical guidelines [4–6]. Aside from offering a target for biopsy and treatment planning, mpMRI provides the unprecedented opportunity to track prostate cancer over time. Prior to the MRI era, surveillance depended heavily upon systematic, non-targeted biopsy. MRI features together with targeted histological data have significantly improved our understanding of individual tumour characteristics and for the first time allow us to prospectively study tumour dynamics.

Novel biomarkers (radiomic, tissue-based and fluidic) may further refine risk stratification and identify tumours where surveillance may be favoured. Genomic, transcriptomic, epigenomic and methylomic analysis has identified vast underlying molecular heterogeneity within prostate cancer foci and suggests that this is unlikely to be a disease where there is a single actionable target [7,8]. Numerous molecular aberrations have, however, been found to be prognostic for disease recurrence, and may represent useful predictors of disease risk [8]. To our knowledge, a longitudinal assessment of molecular changes within a single prostate cancer focus, such as the planned analyses within RECONCILE, has never been performed and remains poorly understood. It is however, known that the molecular landscape of early prostate cancer differs from late stage, metastatic castrate resistant prostate cancer [8]. A longitudinal assessment of prostate cancer molecular heterogeneity over time may therefore help determine the optimal “time to treat” for those who pursue surveillance. Beyond genomic analysis, the immunological environment of a localised prostate tumour is completely unexplored. The dynamics of a related immune response will be explored longitudinally within RECONCILE to further understand this process and its contribution to prostate cancer aetiology and progression.

Specific underlying molecular events may also be captured during radiomic analysis and there is a known association between MRI conspicuity and underlying biological events at the genomic level [9]. Previous work has suggested potential value in the longitudinal evaluation of quantitative parameters extracted from MR images (e.g., the Apparent Diffusion Coefficient–ADC–from diffusion-weighted imaging) [10]. Changes in prostate cancer volume on MRI imaging similarly provides an objective measure of response to treatment or disease progression. Volume measures are used during standard of care surveillance and form a key component of the clinical Prostate Cancer Radiological Estimation of Change in Sequential Evaluation (PRECISE) score which estimates the likelihood of radiological progression in patients on active surveillance for prostate cancer with serial mpMRI [11]. Emerging imaging techniques such as hyperpolarised MRI use carbon-13 enriched metabolites as tracers, in particular 13C-pyruvate, to capture the metabolic activity of tumours and may dramatically improve the sensitivity for detection of aggressive tumours when used alongside traditional mpMRI imaging [12]. mpMRI texture analysis has also shown promise in detection and risk stratification of MR-visible cancers [13]. RECONCILE will provide a platform for each of these imaging techniques to be further evaluated.

#### 1.2 Aims and objectives

RECONCILE (NCT04340245) will identify molecular and radiomic markers associated with clinical, pathological and radiological change in a cohort of localised MRI visible Gleason 3 + 4 tumours (n = 60). Markers will be correlated against diagnostic MRI-targeted histology and oncological outcomes.

The primary objective is to estimate the association of molecular changes with radiological progression (i.e., PRECISE 4 and 5) events of small, localised Gleason 3 + 4 cancer visible on MRI. Secondary and exploratory objectives include opportunity for further analysis of clinical and radiological progression events within the cohort, emerging imaging techniques (radiomic analysis and hyperpolarised MRI sequencing), identification of molecular mechanisms underlying cancer progression events, exploration of localised prostate cancer immune contexture and tumour micro environement and evaluation of participant quality of life scores using validated questionnaires. RECONCILE will provide an opportunity to derive models for prediction of prostate cancer progression risk based on molecular, imaging and histological analysis.

## 2. Design and methods: The RECONCILE study protocol

### 2.1 Study overview

RECONCILE is a single-centre, prospective, longitudinal, observational cohort study of patients recently diagnosed with localised prostate cancer. The study represents level 1b evidence for prognostic and diagnostic studies [14]. This manuscript is based upon study protocol version 2.0 10July2020.

RECONCILE will recruit 60 evaluable participants with a PSA of ≤ 15ng/ml, organ-confined Gleason 3 + 4 cancer (MCCL ≤ 10mm) and an MR concordant lesion (Likert or Prostate Imaging Reporting and Data System—PI-RADS—4 or 5) who have chosen to pursue active surveillance. Participants must have no history of prior prostate cancer treatment or anti-androgen exposure in the preceding 6 months (including 5-alpha reductase inhibitors) to be eligible for enrolment. Full inclusion and exclusion criteria are outlined in Table 1.

**Table 1:** RECONCILE study inclusion and exclusion criteria.

**Inclusion Criteria**
Adult aged 18 years or above
Diagnosed with prostate cancer Overall Gleason score 7 (3+4) within 12 months of entry
Likert or PIRADS score greater than or equal to 4
PSA less than or equal to 15 ng/ml in the last 6 months
Multiparametric MRI concordant with histology
Maximum cancer core length less than or equal to 10mm
Patients on active surveillance
**Exclusion Criteria**
Any contraindication to MRI scans (e.g. metal implants, unmanageable claustrophobia)
Radiological progression on any pre-enrolment MRI prostate following a previous histological diagnosis of Gleason 7 (3+4) prostate cancer
Presence of a pacemaker
Presence of a hip replacement
Any hormonal treatment or inhibitors of 5 alpha-reductase in the previous 6 months
Any previous TURP or other prostate surgery
Previous treatment for prostate cancer
Patients who have previously had sepsis due to a prostate biopsy
Patients receiving concomitant treatment for their cancer
Inability to provide full informed consent (e.g. due to dementia)

Tumour and fluidic samples will be subject to molecular analysis at baseline and one year. All participants will donate blood, urine and prostate tissue at baseline and one year. Prostate tissue will be collected during targeted transperineal prostate biopsy, directed to the MRI lesion of interest. A small subset of participants who are also enrolled in the Prostate Liquid Study (PLiS) (NCT04102904) will be invited to donate an interval semen sample at one year. A baseline semen sample will be shared from PLiS with the patients’ consent. For continuity, multiparametric MRI will be acquired on the same scanner (either 1.5T or 3T / only 3T) at baseline and interval. Radiomic features from standard of care multiparametric MRI will be extrapolated at baseline and one year. Molecular and radiomic markers of disease, and disease progression, will be identified during the final multivariate analysis.

Participants exit the study after 12 months and revert to the NHS standard of care prostate cancer pathway. No further study visits are required. Long-term participant follow-up through national healthcare records will then commence. Longitudinal outcomes (time to metastasis and disease-specific death) will be informed by long-term healthcare data linkage via national health records.

**Fig 1:**
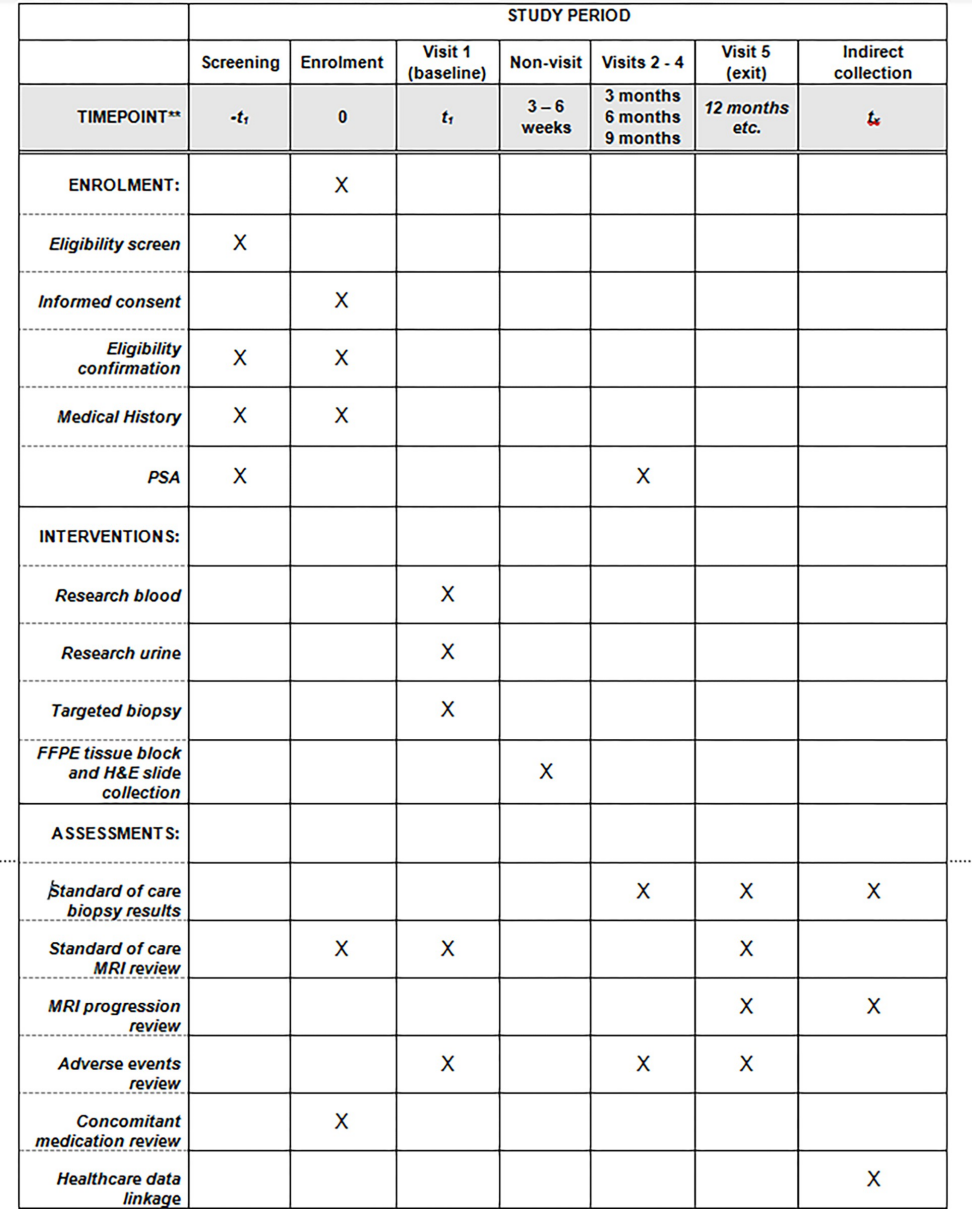
The SPIRIT schedule of enrolment, interventions and assessments for the RECONCILE study.

Participants who are also enrolled to the VALIDATE-PRO Study (NCT05017181) will have the opportunity to share data from their baseline and interval VERDICT, Luminal Imaging and hyperpolarised MRI studies for further analysis within RECONCILE [15–17]. The SPIRIT schedule of enrolment, interventions and assessments summarises study design (Fig 1).

## 3. Objectives

The RECONCILE study will characterise the molecular changes and other factors associated with first identifiable radiological progression event in early Gleason 3 + 4 prostate tumours. Deep phenotyping of the MRI lesion in question will inform novel strategies for improved baseline disease risk stratification.

### 3.1 Primary objective

The primary objective is to estimate the association of molecular changes with radiological progression events of small, localised Gleason 3 + 4 tumours visible on mpMRI.

### 3.2 Secondary objectives

Secondary objectives will assess the impact and associations of radiological tumour progression on a broad set of criteria. Specifically, the secondary objectives will:

*Examine the effect of progression on*:

- Mechanistic events occurring at genetic, epigenetic, transcriptomic and proteomic level
- Imaging features of the MRI lesion in question as well as the rest of the gland
- Digital pathology (cancer and benign tissue)
- Tumour microenvironement
- Immune contexture of prostate cancer and prostate tissue
- Transition to active treatment and any impact upon eligibility for available treatment options
- Patient reported quality of life outcomes
- Recurrence after treatment and progression to metastasis

*Derive*:

- Progression endotypes (cohorts) based on genetic, epigenetic, transcriptomic, and proteomic immune determinants of MRI progression
- A predictive model of progression based on molecular analysis, imaging, histology and clinical data
- Time to radiological progression
- Data to inform existing health economic modelling

*Refine*:

- A molecular index (MoI) of progression derived from analyses of biopsies at baseline and at a 12-month interval
- A model of progression as stochastic, deterministic or a transition between the two at molecular analysis

*Assess*:

- Inter-observer variability of the PRECISE score [11] and MRI lesion segmentation
- Feasibility and acceptability of a novel system of care driven by a demonstrable phenotype at baseline rather than clinical risk estimation

### 3.3 Exploratory objectives

Exploratory objectives will assess any parameters linked to progression, including to invasive prostate cancer treatment and any clinically relevant events such as failure of primary treatment or metastasis. Clinical events will be reviewed by staff who are authorised to access patient medical records during longitudinal follow up.

## 4. Study design

### 4.1 Study entry

**Fig 2:**
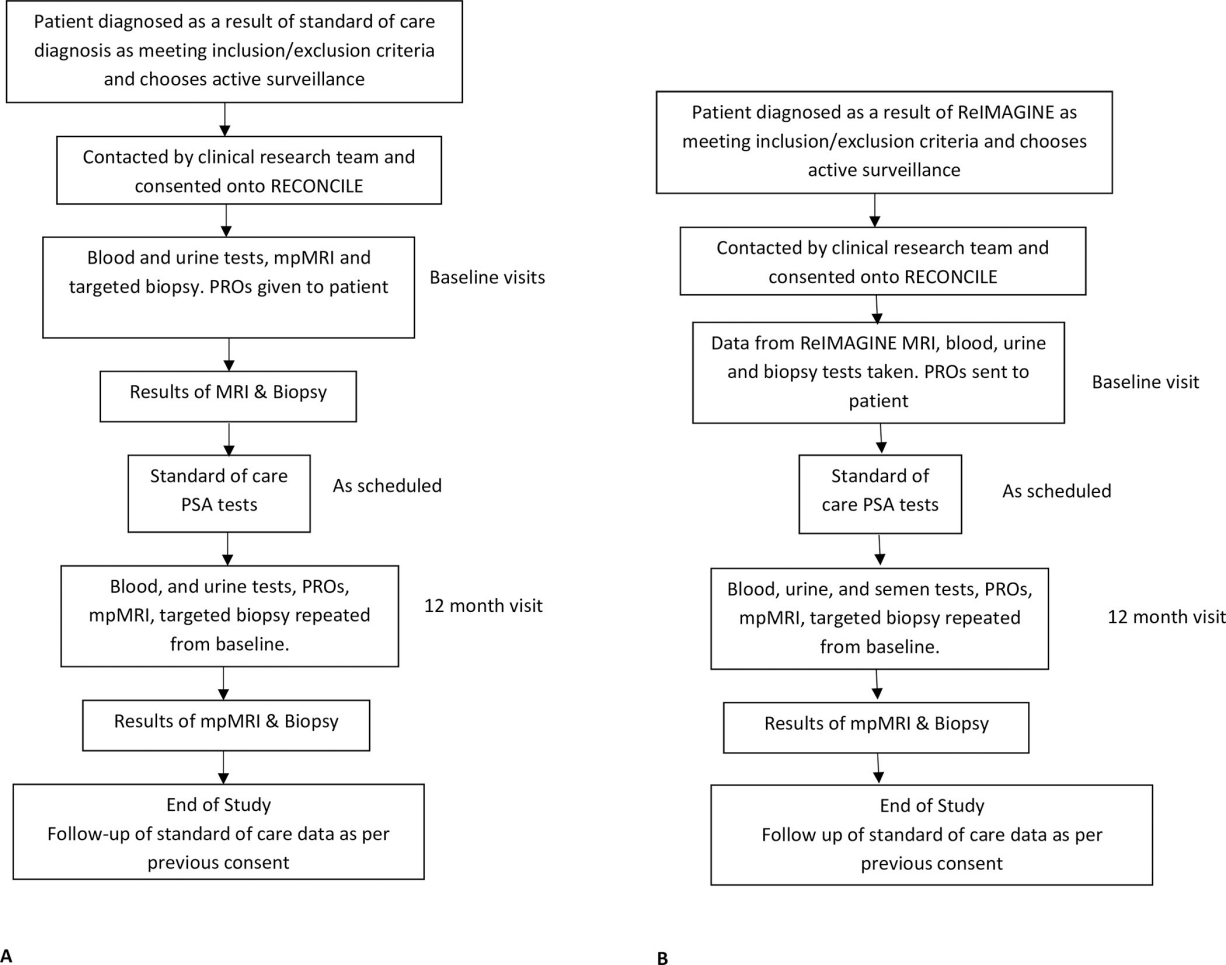
A & B: RECONCILE participant pathway.

Participants may enter RECONCILE following participation in the ReIMAGINE Prostate Cancer Risk Study (NCT04060589) or at the time of tertiary referral for disease re-stratification (Fig 2). The ReIMAGINE Risk Study is running concurrently at site and provides a platform for deeply phenotyping prostate mpMRI lesions (>/ = Likert / PI-RADS 3) through molecular and radiomic analysis [18].

Participants recruited following participation in ReIMAGINE Risk will consent to the sharing of their biological samples and MR imaging with RECONCILE. Therefore, no additional baseline interventions are required for those recruited to RECONCILE from ReIMAGINE. Participants will, however, consent to donation of tissue and MRI data at one year.

An alternative route of entry is at the time of standard of care confirmatory targeted biopsy. This will normally follow a specialist referral to our centre for disease re-stratification and/or management. Such patients must have been diagnosed with Gleason 3 + 4 prostate cancer within 12 months of entry to RECONCILE and meet all other eligibility criteria. Research samples (blood, urine and prostate tissue) will be collected at the time of standard of care confirmatory targeted prostate biopsy. A repeat mpMRI prostate is also required as per local NHS protocol, a copy of which will be used for research. Interval research samples will be collected at one year.

### 4.2 Exposure variable

The main exposure variable is the deep phenotyping of the MRI-visible lesion at baseline and one year. This will be achieved using donation of tissue from additional targeted sampling (using visual-estimation) of the MR-visible lesion of interest harbouring Gleason 3 + 4 disease. A control sample collected from an area of unremarkable tissue on MRI (Likert / PI-RADS v2.1 score 1 or 2) will further inform the characterisation of the MR lesion.

### 4.3 Generation of the population for association between molecular changes and radiological progression

RECONCILE will identify molecular changes associated with radiological progression events in localised Gleason 3 + 4 tumours. Investigators assessing molecular and radiological changes at baseline and one year will be kept blinded to the results of each outcome.

A contingency table will be generated to assess the interrelation between radiological progression and molecular changes. The table will include the following variables: radiological progression (R+), radiological non-progression (R-), Molecular changes (M+), no molecular changes (M-). The contingent for each subgroup to maintain association will be calculated for a set precision to capture significant molecular changes and assess the interaction between molecular change and radiological progression.

### 4.4 Assessment of molecular change

We have designed a molecular analysis strategy that accounts for three potential pitfalls which need to be overcome: tumour content, sampling bias and spatial heterogeneity of the tumour.

#### 4.4.1 Molecular index

We will use next-generation sequencing and expression analyses to derive a quantitative molecular index for every baseline (MolBL) and follow-up (MolFU) biopsy. For each individual patient, the difference in the baseline molecular index between the two baseline biopsy samples (ΔMolBL) will be calculated. This is a measure of intrinsic patient tumour heterogeneity and will identify molecular features which are consistent between lesions, overcoming sampling bias. The relationship of the targeted biopsy sample to the longitudinal axis of the cancer lesion will also be taken into account using multi-slice processing and separate processing of the sample extremities.

We will use the same metrics to generate a MolFU for each patient using per protocol follow-up biopsy samples. We will then compare MolBL and MolFU for every individual patient and extract a ΔMolBL:MolFU score. Patients will be classified as having a molecular progression event if the ΔMolBL:MolFU score is greater than 95% CI of the combined ΔMolBL scores. Researchers generating the molecular index score will be blinded to radiological progression annotations.

#### 4.4.2 Spatial heterogeneity

Spatial heterogeneity within the same tumour will be taken into consideration. As all biopsies will be taken transperineally and directed to the centre of the lesion in axial plan, a biopsy core explores the longitudinal morphometric axis of the cancer. Using a standard biopsy gun, the length of tissue sampled is 17mm long and 16gauge (1.29mm) thick. Patients under active monitoring are not expected to have cancer involvement of the whole biopsy core length. The maximal cancer core length harboured on a single core and percentage of cancer involvement of a core are well established considerations when entering a surveillance programme. These specimens will reflect the centre, the periphery and the immediately adjacent tissue of the cancer.

The biopsy sample will be analysed as a multi-slice process. The first slice will undergo standard Haematoxylin and Eosin staining. The extremity of the cancer observed within the core will be analysed separately.

### 4.5 Assessment of radiological progression

Radiological progression will be defined using the PRECISE score for which consensus has been published [11]. A PRECISE score of 4 or 5 will define radiological progression, a PRECISE score of 1, 2 or 3 will define radiological regression / stability ie non radiological progressors.

#### 4.5.1 Inter-observer variability

To compensate for inter-observer variability, double reading of MRI will be performed. In case of discordance between radiologists regarding the binary outcome of progression (PRECISE 4 and 5) vs non progression (PRECISE 1, 2 and 3), a third, independent reviewer will determine the final result in disagreement cases. All 3 radiologists will be involved in reporting RECONCILE imaging. A third party, not involved in the radiology assessment, will be unblinded to the radiology report and if necessary, will reallocate indeterminate MRIs for reporting. The 3rd radiologist will be blinded of their 3rd reviewer role.

#### 4.5.2 Blinding from biopsy results

Radiologists reporting the second mpMRI will be kept blinded from the results of the second set of biopsies, performed at 1 year from baseline.

#### 4.5.3 Consensus meeting

A joint consensus meeting with the urologist performing the biopsies, trial radiologist and pathologist will review the consistency of radiological progression and standard of care biopsy results. Sampling effect will be taken into account. Blinding to the results from molecular analysis will be maintained during this process.

#### 4.5.4 Final determination of radiological progressors

The final number of radiological progressors and non-radiological progressors will be established using the double reported MRIs and consensus meeting outcome if needed.

### 4.6 Data reconciliation

To minimise bias, radiological data identifying progressors and non-progressors will be blinded from those performing the concurrent molecular analysis.

#### 4.6.1 Precision for detection of significant changes in the molecular characteristics

Data from radiological progressors and non-progressors will allow us to populate the contingency table. We will statistically derive the minimum precision for significance of changes in molecular characteristics needed to maintain the association between radiological and molecular progression.

#### 4.6.2 Determination of molecular progressors

Precision will be applied to changes in molecular characteristics to determine significant variation between baseline and follow up molecular indices for every studied parameter derived from the molecular analysis.

### 4.7 Patient and public involvement

The RECONCILE study was originally conceived as a work stream of the ReIMAGINE Risk study and was included in the patient and public involvement (PPI) consultation that took place for this. The study design (including the number of visits and outcome measures) and all patient facing documents have been reviewed by two PPI committees, first the ReIMAGINE PPI committee consisting of patients and members of the wider public who have been affected by, or have experience of, prostate cancer and second, the South East London Consumer Research Panel for Cancer.

### 4.8 Phase of trial

RECONCILE is a single site study which opened to recruitment in July 2020 (n = 62). So far, 10 participants have been identified as non-evaluable. A replacement will be recruited for each non-evaluable case. Recruitment is expected to complete in July 2022. 37 participants have donated baseline samples, and 5 participants have completed active study follow up.

## 5. Study schedule

### 5.1 Screening and recruitment

Potential participants will be identified at the time of referral for tertiary multidisciplinary team review, during routine outpatient clinic, or following participation in the ReIMAGINE Risk study. Relevant NHS multidisciplinary team meetings (MDT) and recruitment logs to the ReIMAGINE Risk study will be screened weekly by study staff for potential participants. All eligible participants will be offered enrolment.

The first approach of eligible participants will take place over the telephone, or in an NHS clinic. A participant information leaflet (PIL) and other relevant study documentation will be provided to interested patients who meet study inclusion and exclusion criteria (Table 1). Consent is written and can be given over the telephone (with relevant signed documents returned by post) or during a face to face clinical visit.

All those approached or considered for recruitment will be recorded on the study screening log. Participants will consent to sharing of data from ReIMAGINE and PLiS (where applicable), donation of prostate tissue cores, blood, urine (and semen for those enrolled in the PLiS study), and healthcare data linkage.

### 5.2 Study visits

**Fig 3:**
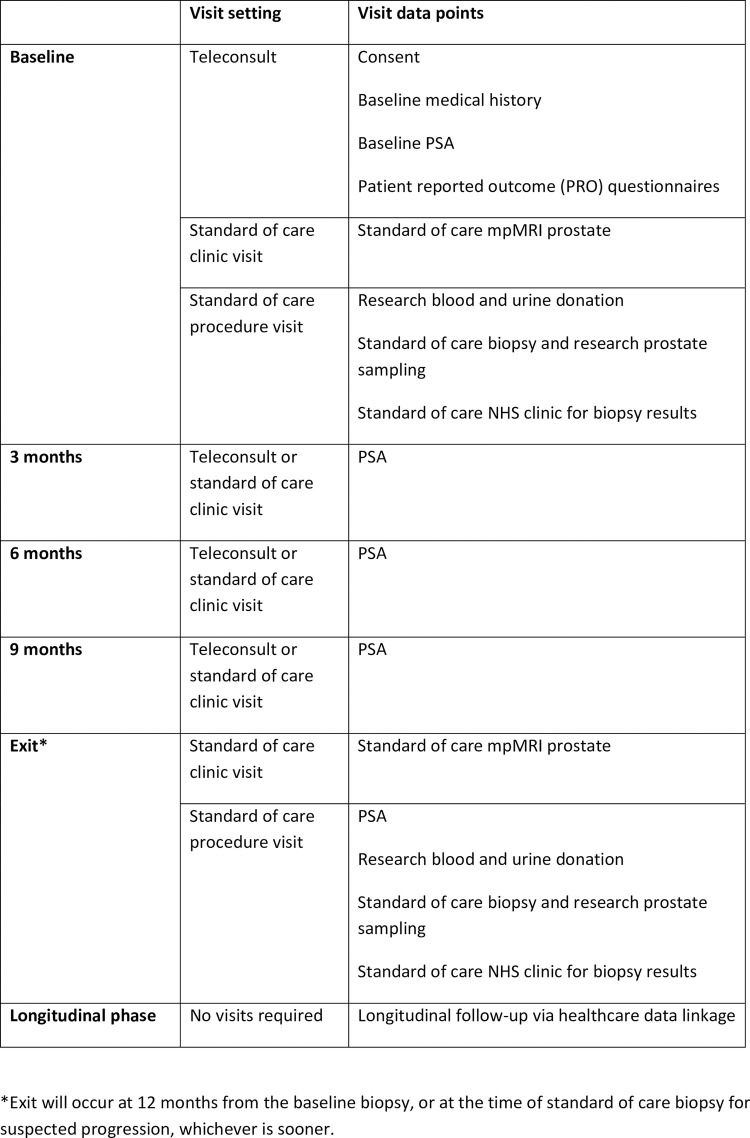
Schedule of RECONCILE study visits.

Study encounters will take place over a 12-month period and do not incur any additional visits beyond standard of care active surveillance (Fig 3).

*Visit 1 (baseline)*

The baseline visit consists of two encounters: a standard of care MRI prostate and a subsequent tissue donation visit at the time of standard of care NHS prostate biopsy. At the biopsy encounter, research blood, urine and prostate tissue will be collected.

Research data including baseline medical history and patient reported outcome (PRO) questionnaires will be collected on or before the standard of care biopsy visit. Baseline medical history review may take place over the telephone.

Participants enrolling to RECONCILE following participation in the ReIMAGINE Risk or PLiS studies will not be required to donate research blood, urine or prostate tissue at baseline. Instead they will consent to data and sample sharing from ReIMAGINE Risk or PLiS to RECONCILE.

*Follow-up PSA visits (3, 6 and 9 months)*

Following the baseline visit, clinical results will be provided in a standard of care NHS prostate cancer clinic. If active surveillance remains a suitable management option, the participant will proceed to interval 3 monthly PSA testing and clinic review as per national guidance. PSA kinetic data will be collected prospectively for research and entered into the study database.

*Interval (exit) visit (12 months)*

Interval (exit) investigations will take place 12 months after baseline tissue collection and consists of two standard of care encounters: an MRI prostate visit and subsequent targeted transperineal prostate biopsy. Again, research blood, urine and prostate tissue will be collected during the standard of care biopsy visit. PRO questionnaires will be posted to participants ahead of the biopsy visit and can be returned to study staff either by post or in person. Participants enrolled in the PLiS study will be invited to donate a semen sample at the time of study completion. This will be collected at home and returned to the study laboratory by post.

*Suspected progression*

Interval investigations, and exit from the study, may occur prior to the 12-month mark if there is suspicion of progression during PSA follow up. As is usual practice, standard of care mpMRI will be expedited and exit research samples collected (PROs, blood, urine, prostate tissue +/- semen) should the patient proceed to standard of care prostate biopsy.

*Non-visit*

Access to standard of care diagnostic biopsy tissue will be sought once the NHS centre has completed all necessary diagnostic evaluation. Formalin-fixed paraffin-embedded (FFPE) tissue blocks and haematoxylin and eosin (H&E) stained slides will be requested by RECONCILE study staff and anonymised H&E slides will be scanned using high resolution scanners and uploaded to the RECONCILE digital pathology database. Slices from standard of care FFPE tissue blocks will be collected in line with study standard of care operating procedures before returning all slides and blocks to the relevant NHS pathology department.

*Analyses*

Blood components (white blood cells, plasma, serum), urine, tissue and semen will undergo molecular analysis including genomic, mRNA expression and protein studies. These will include, but are not limited to, next-generation sequencing, whole-genome micro-array expression profiling, immunohistochemistry and immunofluorescence and ELISA tests.

*Long-term healthcare data linkage*

Consent will be sought to obtain long-term patient outcomes from national health records such as the Office for National Statistics, NHS Digital, Public health England and other applicable NHS information systems or national databases.

### 5.3 Definition of end of study

Following the collection of research samples (PROs, semen for PLiS participants, blood, urine and prostate tissue) at the exit visit men will revert to standard of care NHS follow up. No further study visits are required. This visit completes the end of the first phase of the RECONCILE study. Men will undergo study follow up for 4 to 8 weeks after the biopsy for pathology results to be collected.

Participants then transition to longitudinal follow-up via healthcare data linkage. This represents the second phase of the study for which no face-to-face visits are required. For the second phase of the study, the end of the study is defined as database lock.

## 6. Study interventions

RECONCILE will deeply phenotype MRI lesions of interest at baseline and one year. This will be achieved using additional targeted sampling (using visual-estimation) of the MR-visible lesion harbouring Gleason 3 + 4 cancer. Clinical (PRO and PMH), radiomic and fluidic biomarker (blood, urine, semen) data and samples will also be collected at baseline and exit.

### 6.1 Patient reported outcome questionnaires

Participants will be asked to complete three prostate cancer specific health-related quality of life and symptom severity questionnaires at the time of baseline and exit visits, these include the:

- Expanded Prostate Cancer Index Composite-26 (EPIC-26)
- European Organisation for Research and Treatment of Cancer Quality of Life Questionnaire-C30 (EORTC QLQ-C30)
- Memorial Anxiety Scale for Prostate Cancer (MAX-PC)

Participants’ height, weight, ethnicity, medical history and family history of prostate cancer will be recorded at the time of enrolment.

### 6.2 MRI databank

Multiparametric MRI will be performed as standard of care [4]. Reporting will be according to study SOPs and include Likert and PI-RADS scales documented within a structured electronic case report form (eCRF). Lesions, and the prostate itself, will be contoured and segmented prior to upload to the study image repository, hosted by UCL.

### 6.3 Blood sample collection

Between 50 and 100ml of blood will be collected from each consenting study participant by a trained phlebotomist or RECONCILE Clinical Trial Practitioner during the baseline and exit visits. Venepuncture and sample collection will be performed in accordance with the RECONCILE Blood Sampling SOP. The required samples are outlined in Table 2. Whole blood samples will be processed by a trial Laboratory Technician or Clinical Trial Practitioner at the study affiliated UCL laboratory. Details of sample processing are outlined in Appendix I.

**Table 2:** Blood samples to be collected during the RECONCILE study baseline and exit visits.

Order of draw (by collection tube)	Number of tubes to be collected	Volume per tube (ml)
EDTA	1	10
SST	1	8.5
Streck^TM^	1	10
EDTA	1	10
PAXgene® Blood RNA	1	2.5
EDTA	1	10
Extra EDTA (optional)	0–6	10

Between 50 and 100 ml of blood will be collected from each consenting study participant by a trained phlebotomist or RECONCILE Clinical Trial Practitioner during visit 1 and once again at exit from the study. Venepuncture and sample collection will be performed in accordance with the RECONCILE Blood Sampling SOP. The required samples (to be collected both at baseline and once again at exit) and order of draw is outlined in Table 2.

### 6.4 Urine sample collection

An early morning urine sample will be collected in line with the study SOP by study practitioners during the baseline and exit visit. Urine collection will always take place prior to prostate biopsy. The pseudonymised samples will be transferred to a study affiliated UCL laboratory and logged within the study FreezerPro database. Both unspun and centrifuged urine will be stored within the study biobank. Spun samples will be centrifuged using a one-step process (3300g for 20 mins, with a break, at room temperature). Both centrifuged and unspun urine will be stored at -80C as will the residual cell pellet.

### 6.5 Prostate tissue collection

Consenting patients will donate three additional cores of prostate tissue at the time of their baseline standard of care prostate biopsy. At exit, participants will donate a further 3 biopsy tissue cores, or five additional cores if there is a new lesion on mpMRI. Cores 4 and 5 will be collected from the highest scoring, greatest volume new lesion.

We do not anticipate that collection of additional tissue cores for research will increase the likelihood of side effects or adverse events relative to standard of care biopsy alone. The risks and adverse outcomes of prostate biopsy quoted within the RECONCILE patient information sheet are outlined in Table 3. Research cores will be targeted using visual-estimation to areas of interest identified on pre-biopsy mpMRI. Research cores will only be attempted after all required standard of care diagnostic biopsies have been collected.

**Table 3:** Side effect profile of a targeted transperineal prostate biopsy as stated in the RECONCILE study patient information sheet.

Side effect	Proportion of men	Duration
Pain/discomfort in back passage	Almost all	Temporary for 1–2 days
Burning when passing urine	Almost all men	Self-resolving, 1–3 days
Bloody Urine	Almost all	Self-resolving, 1–7 days
Bloody Sperm	Almost all	Lasting up to 3 months
Poor erections	1–2 in 100	Temporary for 1–6 weeks
Infection of skin/urine	1–2 in 100	7 days with treatment with antibiotics
Infection of skin/urine needing admission and intravenous antibiotics	Less than 1 in 500	7–14 days with treatment with antibiotics. Rarely up to 28 days of antibiotics after leaving hospital.
Difficulty passing urine requiring catheter placement for up to a week. A catheter is a soft plastic tube placed into the bladder to drain urine.	1 in 100	3–7 days
Bruising of skin	Almost all	Self-resolving, 7–14 days
Bruising spread to scrotum	1 in 100	Self-resolving, 7–28 days

All cores will be collected in line with the study Tissue Collection SOP. The first and second research cores will be collected from the centre of the lesion with the highest Likert / PIRADS score and greatest volume (lesion 1). Lesion volume will be determined by MRI reporting.

The third research core will be collected from an area of non-suspicious (Likert / PIRADS score 1 or 2) tissue. Should new MRI lesions be visible at the time of exit from the study (minimum Likert / PIRADS score 4), two additional tissue cores (Cores 4 and 5) may be collected from the centre of the new highest scoring, greatest volume lesion. Table 4 summarises the location from which to collect each prostate biopsy core.

**Table 4:** Prostate tissue sampling locations for the RECONCILE study.

Research Core	Lesion to target	Area within lesion to target	Casette colour
Core 1	Lesion with highest PIRADS / Likert score and greatest volume (lesion 1)	Centre of the lesion	Pink
Core 2	Same lesion as above (lesion 1)	Centre of lesion 1	Green
Core 3	Contralateral nonsuspicious area	Any non-suspicious radiological area, either contralateral lobe or TZ if bilateral lesions or diffuse changes in bilateral lobes	Yellow
Core 4 (optional)	New radiological lesion with the highest PIRADS/LIKERT score at interval assessment (lesion 2)	Centre of lesion 2	Any colour, labelled as Core 4
Core 5 (optional)	New radiological lesion with the highest PIRADS/LIKERT score at interval assessment (lesion 2)	Centre of lesion 2	Any colour, labelled as Core 5

**Fig 4:**
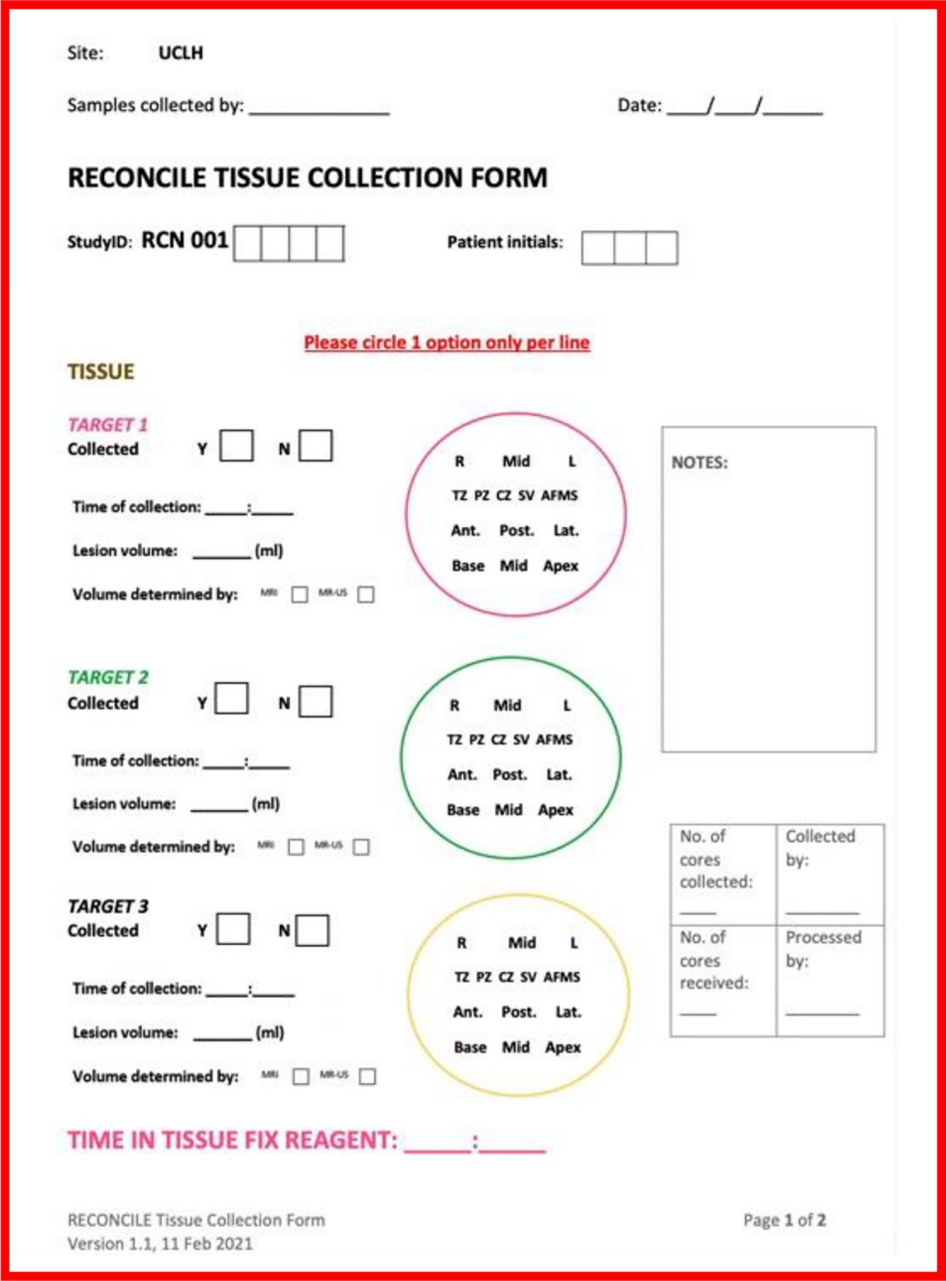
RECONCILE study Tissue Collection Form (eCRF).

The location of each prostate core will be recorded within a study CRF (Fig 4) and each core placed within a colour coded pathology cassette before being placed within PAXgene® tissue fixative. Samples will be fixed in PAXgene® Tissue Fix reagent for at least 2–24 hours. Cores are then treated with PAXgene® Tissue Stabalizer concentrate and ethanol for at least 2 hours. All pseudonymised samples will be logged to the FreezerPro database and stored at -80°C following stabiliser treatment. All biological samples are processed and stored in line with the RECONCILE Laboratory Manual.

### 6.6 Semen sample collection

PLiS participants will be invited to donate a semen sample at the time of their exit visit. The sample will be collected at home (ahead of prostate biopsy) and returned to the study affiliated laboratory by post or courier. Their baseline comparator sample will be shared from the PLiS study which collects pre-biopsy semen samples in prostate biopsy-naive men.

### 6.7 Formalin-Fixed Paraffin Embedded (FFPE) tissue blocks and haematoxylin and eosin (H&E) slide collection

In addition to the prostate tissue cores collected for research, standard of care diagnostic pathology FFPE tissue blocks and H&E slides will be requested from the participating centre once all diagnostic evaluation is complete. Pseudonymised H&E slides will be scanned using the Hamamatsu Nanozoomer S360 high-throughput digital slide scanner at a resolution of 0.23 μm/pixel. Digitalised, pseudonymised slides will be stored within a dedicated RECONCILE database held securely on UCL servers.

Digitalisation will facilitate computer-based assessment of morphological features. RECONCILE tissue technicians will also collect tissue slices from standard of care FFPE tissue blocks to provide additional tissue for analysis. All FFPE tissue blocks and H&E slides will be returned to the relevant NHS pathology department. All processes will be conducted in accordance with study SOPs.

### 6.8 Standard of care PSA levels

Participants will undergo standard of care monitoring with three-monthly PSA blood testing at the recruiting NHS site. Each standard of care PSA value reported during active participation in the study will be entered into the study database.

## 7. Outcomes

### 7.1 Primary outcome

The primary outcome of the RECONCILE study is the proportion of concordant pairs of molecular and radiological progressors at exit from active follow up (usually at 12 months).

### 7.2 Secondary outcomes

Secondary objectives span imaging, pathology, molecular and clinical features and include:

*Imaging*

- Time to radiological progression on standard of care MRI scan (i.e. evidence of MRI change either at planned standard of care MRI scan at one year from baseline, or on an expedited standard of care prior to this
- Quantitative and qualitative assessment of MRI lesion(s) imaging characteristics
- Quantitative imaging features (MRI and derivative) of the MRI lesion(s), a radiological progression ring (region of interest of the periphery within the MRI lesion at follow up which did not harbour abnormal signal at baseline), if present, and the rest of the prostate at different time points
- Qualitative imaging features (MRI and derivative) of MRI lesion(s), a radiological progression ring (if present) and the rest of the prostate at different time points
- Quantitative imaging characteristics of MRI lesion(s), a radiological progression ring (if present) and the rest of the prostate at different time points and stratification according to radiological progression vs non progression

*Histology*

- Qualitative and quantitative histologic composition of cancer lesions and their surrounding tissue
- Histological heterogeneity of cancer, both qualitatively and quantitatively

*Molecular analysis*

- The molecular index (as defined in section 4.4.1 of this protocol) of the cancer lesion in question, as well as the surrounding peritumoral and normal tissue
- Qualitative and quantitative analysis of urinary and seminal biomarkers
- Qualitative and quantitative analysis of inflammatory infiltrate in the cancer lesion, peritumoral and normal tissue
- Analysis of blood-based biomarkers related to immune surveillance and inflammatory cytokines
- Qualitative and quantitative analysis of immune related pathways

*Clinical data*

- Proportion of patients who transition to active treatment, stratified according to the timing and nature of treatment
- Proportion of patients eligible for each treatment option at baseline and follow-up
- Rate of metastasis at specified time points during follow up
- Concordance rate between histological and radiological progression
- Concordance of PRECISE score reporting among study radiologists
- Patient reported outcomes at different time points using prostate cancer-specific health-related quality of life and symptom severity scores using the Expanded Prostate Cancer Index Composite-26 (EPIC-26), the European Organization for Research and Treatment of Cancer-Quality of Life Questionnaire-C30 (EORTC QLQ-C30) and the Memorial Anxiety Scale for Prostate Cancer (MAX-PC)

### 8. Statistical analysis

The primary statistical analysis will be to estimate the association of match‐paired binary responses (molecular and radiological progressors) since the results of the two tests are not independent. For McNemar’s test of paired proportions using the Miettinen normal approximation with a two‐sided significance level of 0.05, a sample size of 60 achieves a power of at least 0.8 when the discordant proportions are 0.05 (5%) and 0.25 (25%) of the total number. The ratio of progressors will inform the precision of the IC for the molecular analysis. Study results will be shared in peer reviewed journal articles.

## 9. Ethical considerations

The study abides by the principles of the Declaration of Helsinki and received regulatory approval from the Regional Ethics Committee (London–Stanmore 20/LO/0366, 15 April 2020). RECONCILE is published on clinicaltrials.gov (ClinicalTrials.gov identifier: NCT04340245).

## 10 Discussion and limitations

RECONCILE (NCT04340245) will identify molecular markers associated with radiological progression events in a cohort of localised, newly diagnosed Gleason 3 + 4 tumours. Each tumour will be deeply characterised using MRI-targeted histology within a standard of care pathway. Fluidic (blood, urine, semen) and radiomic markers of change will also be assessed. Markers will be correlated against standard of care MRI-targeted histology and long-term oncological outcomes extracted from national health records. By characterising the first detectable radiological progression events within this cohort, and defining image-based progressor and non-progressor cohorts, we may accelerate baseline risk stratification for a significant proportion of men.

This is particularly relevant to the Gleason 3 + 4 cohort which forms the bulk of incidentally detected prostate cancers [19]. Prospective and retrospective data sets suggest that initial active surveillance is likely to be a satisfactory option for men with localised, low-intermediate grade, prostate tumours [3,20]. This is particularly pertinent since radical treatment, the side-effect profile of which is well documented, remains an option for this cohort in several key clinical guidance [4]. Further stratification of the cohort is necessary to spare premature, or altogether unnecessary, intervention.

mpMRI has redefined localised prostate cancer diagnostics and imaging-led surveillance strategies. Novel tests such as radiomic, tissue-based and fluidic biomarkers may further refine risk stratification at baseline and guide the need for intervention by predicting risk of progression. RECONCILE represents the first efforts to assess this prospectively in a cohort of Gleason 3 + 4 tumours. Numerous molecular aberrations are known to be prognostic for prostate cancer recurrence and differ between early, and advanced disease. Such markers may represent predictors of progression risk at baseline [8]. Novel imaging techniques also show promise in the detection and risk stratification of localised prostate cancers. An integrated approach using clinical, molecular (whether fluidic or tissue-based) and imaging analysis may therefore provide more accurate risk assessment and distinguish “progressors” from tumours less likely to progress and prove to be more accurate than histopathological grade–the current gold standard.

### 10.1 Limitations

The RECONCILE protocol has some potential limitations. First, biomarkers of progression will only be validated against known Gleason 3 + 4 tumours. Further studies would need to assess potential biomarkers of progression against further grade groups in order to develop more clinically useful tests. The imaging and biomarker platform provided by the ReIMAGINE Risk study (NCT04060589) may achieve this in part but does not assess radiological progression events specifically.

Second, the study does not contain a specific control group. Rather, control tissue is collected from an area of Likert / PIRADS score less than or equal to 2 during each research biopsy. This step mitigates against the absence of a normal MRI cohort, accounts for molecular heterogeneity within normal gland tissue and will act as a control. Molecular profiling and long term follow up of localised and advanced prostate cancers undertaken by other groups will provide a comparator for the deeply characterised Gleason 3 + 4 cohort studied within RECONCILE and may add context to our results.

Third, many patients referred for secondary care assessment and therefore captured within the study population are referred because of a raised PSA level. Further work may be required to assess the applicability of findings to a cohort of men with a normal PSA. A small subset of men within RECONCILE will be recruited following participation within the ReIMAGINE Prostate Cancer Screening study [21]. ReIMAGINE Screening has been designed to assess the feasibility of bi-parametric prostate MRI as a screening tool for prostate cancer. Patients may screen positive on the basis of either, or both, MRI positivity and raised PSA density. This cohort may broaden the spectrum of patients recruited to RECONCILE to include some participants with a normal PSA.

## 11. Conclusion

Intermediate risk, localised prostate cancers are amenable to a range of management strategies, including active surveillance, focal ablation, radical prostatectomy and radiotherapy. UK clinical guidance supports the use of active surveillance as a strategy to reduce overtreatment of low-intermediate risk disease.

RECONCILE (NCT04340245) will identify molecular and radiomic markers associated with clinical and radiological progression events in a cohort of localised, newly diagnosed Gleason 3 + 4 tumours which have been deeply characterised with MRI-targeted histology within a standard of care pathway. Markers will be correlated against standard of care MRI-targeted histology and oncological outcomes.

## Supporting information

S1 File SPIRIT checklist. (DOC)

S2 File Appendix I. Blood sample processing.
(DOCX)

S3 File Appendix II. Material and sample storage.
(DOCX)

S4 File Withdrawal of participants. (DOCX)

## Abbreviations

eCRF: Electronic Case Report Form
EU: European Union
FFPE: formalin-fixed paraffin-embedded
H&E: haematoxylin and eosin
HTA: Human Tissue Authority
MRI: magnetic resonance imaging
MTA: material transfer agreement
mpMRI: multiparametric magnetic resonance imaging
NHS: National Health Service
PCa: prostate cancer
PSA: prostate specific antigen
REC: Research Ethics Committee
SOP: Standard Operating Procedure
TRUS: Transrectal Ultrasound
UCL: University College London
