# Supplementary material for: The RECONCILE study protocol: Exploiting image-based risk stratification in early prostate cancer to discriminate progressors from non-progressors (RECONCILE)
Source: PLoS One. 2024 Oct 17;19(10):e0295994. doi: 10.1371/journal.pone.0295994 (PMC11486392; doi:10.1371/journal.pone.0295994)
Supplement: S2 File — Blood sample processing. (DOCX) [file pone.0295994.s002.docx]

**S2 File. Appendix I. Blood sample processing**

*EDTA processing*

Samples collected in EDTA vials will be centrifuged in a two-spin process at 4^○^C: 1600g for 15 minutes, followed by 3000g for 10 minutes (plasma only).

*SST processing*

Blood will be allowed to clot at room temperature for at least 30 minutes after sample collection, refrigerated and centrifuged at 1600g for 10 minutes at 4^○^C (with a break) no later than 4 hours after sample collection.

*Strek^TM^ processing*

Strek^TM^ vial samples will be centrifuged in a two-spin process at room temperature: 1600g for 15 minutes, followed by 3000g for 10 minutes.

*PAXgene® Blood RNA*

Blood samples will be stored in PAXgene® Blood RNA vials for at least two hours after collection and stored in the original collection tubes at -20^○^C for 24 – 48 hours followed by long-term storage at -80^○^C.
