# Supplementary material for: The RECONCILE study protocol: Exploiting image-based risk stratification in early prostate cancer to discriminate progressors from non-progressors (RECONCILE)
Source: PLoS One. 2024 Oct 17;19(10):e0295994. doi: 10.1371/journal.pone.0295994 (PMC11486392; doi:10.1371/journal.pone.0295994)
Supplement: S3 File — Material and sample storage. (DOCX) [file pone.0295994.s003.docx]

**S3 File. Appendix II. Material and sample storage**

Blood, urine, semen and prostate tissue will be collected from RECONCILE study participants in accordance with the patient consent form and patient information sheet. Samples will be processed, stored and disposed in accordance with all applicable legal and regulatory requirements, including the Human Tissue Act 2004 and any amendments thereafter.

Access to any human samples requires a request and approval by the biological research group responsible for the maintenance of scientific rigour and prioritisation of material. Samples are not to be processed and/or transferred other than in accordance with the patients’ consent. After ethics approval for the study has expired biological samples will be disposed of in accordance with the Human Tissue Act 2004 and any amendments thereto or transferred to a licensed tissue bank.

Samples will be identified by the use of pseudo-anonymised study ID numbers.
