# Supplementary material for: The RECONCILE study protocol: Exploiting image-based risk stratification in early prostate cancer to discriminate progressors from non-progressors (RECONCILE)
Source: PLoS One. 2024 Oct 17;19(10):e0295994. doi: 10.1371/journal.pone.0295994 (PMC11486392; doi:10.1371/journal.pone.0295994)
Supplement: S4 File — (DOCX) [file pone.0295994.s004.docx]

**S4: Appendix III: Discontinuation/withdrawal of participants**

*Early Termination*

Patients will be withdrawn from the study if their baseline confirmatory biopsy following recruitment to RECONCILE reveals prostate cancer that does not meet study inclusion criteria. A further patient will then be recruited in their place to ensure that we achieve recruitment of a total of 60 eligible participants to RECONCILE.

*Early Completion*

If there is suspicion of prostate cancer progression during RECONCILE the patient in question will undergo full standard of care re-assessment of their disease. Often the first sign of suspected progression is a rise in PSA level. Should this occur, we will repeat the PSA in the first instance. If a rise in PSA is confirmed and significant the patient will have an expedited high-quality mpMRI prostate. If the mpMRI does not raise the need for a repeat biopsy, and the patient continues on active surveillance, the patient can remain in the study and complete the planned investigations at one year from enrolment. Although this mpMRI is not a research study, we will request access to the results within the medical record.

If a prostate biopsy is required following a re-assessment mpMRI the patient will exit the study after the required biopsy. We will request exit tissue donation (semen from patients enrolled in the PLiS study, blood, urine and up to 5 additional research prostate tissue cores) at the time of this biopsy. The patient will then exit RECONCILE. No further patients will be recruited to replace this cohort of patients who exit RECONCILE prematurely due to early trial completion.

*Further withdrawal of participants*

In providing full informed consent to participate in the study, participants are consenting to data collection, screening assessments, sample donation, follow-up and healthcare data linkage.

A protocol deviation form will be completed if participants are unable to provide the following samples at either baseline or one year:

• <10ml blood

• <10ml urine

• <1 prostate core

• an mpMRI prostate

A participant may be withdrawn from the study whenever continued participation is no longer in the participant’s best interests, but the reason for doing so must be recorded. Reasons for discontinuing the study may include:

• Intercurrent illness

• Patient withdrawal of consent

• Adverse events rendering the patient unsuitable for further mpMRI or tissue donation

• Loss of the ability to provide ongoing consent

• Persistent non-compliance to protocol requirements

The decision to withdraw a participant from the study will be recorded in the CRF and medical notes. If a participant states that they do not wish to contribute further data to the study their decision must be respected and recorded in the CRF and medical notes. Information obtained prior to patient withdrawal from the trial will be kept. This will be held in line with the Data Protection Act (2018) and General Data Protection Regulations (GDPR).

The trial team will ensure that any patient who exits the trial under any circumstance will be followed up in an appropriate standard of care NHS prostate cancer clinic or equivalent.

If the participant is withdrawn due to an adverse event, the Investigator will arrange to follow up the event for the appropriate time.
